# A T cell–myeloid IL-10 axis regulates pathogenic IFN-γ–dependent immunity in a mouse model of type 2–low asthma

**DOI:** 10.1016/j.jaci.2019.08.006

**Published:** 2020-02

**Authors:** William J. Branchett, Helen Stölting, Robert A. Oliver, Simone A. Walker, Franz Puttur, Lisa G. Gregory, Leona Gabryšová, Mark S. Wilson, Anne O'Garra, Clare M. Lloyd

**Affiliations:** a National Heart and Lung Institute, Imperial College London, London, United Kingdom; b Asthma UK Centre in Allergic Mechanisms of Asthma, Imperial College London, London, United Kingdom; c Immunoregulation and Infection Laboratory, Francis Crick Institute, London, United Kingdom; d Allergy and Anti-Helminth Immunity Laboratory, Francis Crick Institute, London, United Kingdom

**Keywords:** Severe asthma, type 2–low asthma, IL-10, immune regulation, T cell, macrophage, dendritic cell, IFN-γ, AAD, Allergic airway disease, AHR, Airway hyperresponsiveness, AM, Airway macrophage, APC, Antigen-presenting cell, BAL, Bronchoalveolar lavage, cDC2, Type 2 conventional dendritic cell, cRPMI, Complete RPMI, Ct, Threshold cycle, DC, Dendritic cell, FoxP3, Forkhead box P3, HDM, House dust mite, IL-10Rα, IL-10 receptor α, IM, Interstitial macrophage, IMM, Inflammatory monocyte and macrophage, mLN, Mediastinal lymph node, moDC, Monocyte-derived dendritic cell, PAS, Periodic acid–Schiff, PMA, Phorbol 12-myristate 13-acetate, T1, Type 1, T2, Type 2, T17, Type 17, Teff, Effector T, Treg, Regulatory T

## Abstract

**Background:**

Although originally defined as a type 2 (T2) immune-mediated condition, non-T2 cytokines, such as IFN-γ and IL-17A, have been implicated in asthma pathogenesis, particularly in patients with severe disease. IL-10 regulates T_H_ cell phenotypes and can dampen T2 immunity to allergens, but its functions in controlling non-T2 cytokine responses in asthmatic patients are unclear.

**Objective:**

We sought to determine how IL-10 regulates the balance of T_H_ cell responses to inhaled allergen.

**Methods:**

Allergic airway disease was induced in wild-type, IL-10 reporter, and conditional IL-10 or IL-10 receptor α (IL-10Rα) knockout mice by means of repeated intranasal administration of house dust mite (HDM). IL-10 and IFN-γ signaling were disrupted by using blocking antibodies.

**Results:**

Repeated HDM inhalation induced a mixed IL-13/IL-17A response and accumulation of IL-10–producing forkhead box P3–negative effector CD4^+^ T cells in the lungs. Ablation of T cell–derived IL-10 increased the IFN-γ and IL-17A response to HDM, reducing IL-13 levels and airway eosinophilia without affecting IgE levels or airway hyperresponsiveness. The increased IFN-γ response could be recapitulated by IL-10Rα deletion in CD11c^+^ myeloid cells or local IL-10Rα blockade. Disruption of the T cell–myeloid IL-10 axis resulted in increased pulmonary monocyte–derived dendritic cell numbers and increased IFN-γ–dependent expression of CXCR3 ligands by airway macrophages, which is suggestive of a feedforward loop of T_H_1 cell recruitment. Augmented IFN-γ responses in the HDM allergic airway disease model were accompanied by increased disruption of airway epithelium, which was reversed by therapeutic blockade of IFN-γ.

**Conclusions:**

IL-10 from effector T cells signals to CD11c^+^ myeloid cells to suppress an atypical and pathogenic IFN-γ response to inhaled HDM.

## Graphical abstract

**Figure:**
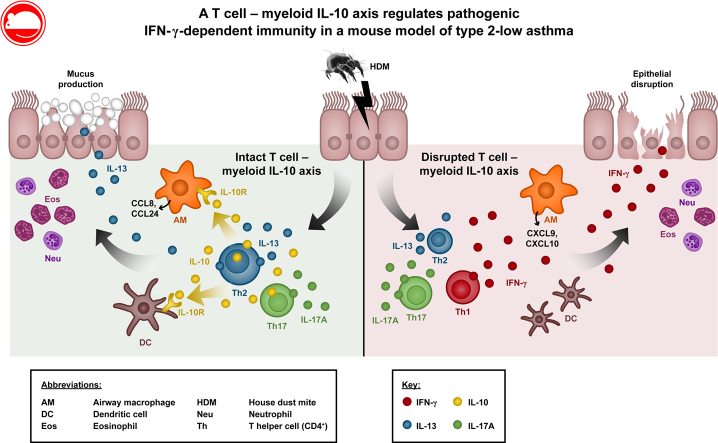

Asthma comprises a broad spectrum of clinical phenotypes with diverse and incompletely understood causes.^1^, ^2^ Dissection of mechanisms in patients with severe asthma is of particular importance because these patients are inadequately treated with conventional therapy regimens and contribute disproportionally to health care costs.^1^ Allergic asthma is typically a type 2 (T2) immune-mediated condition characterized by IL-4, IL-5, and IL-13 production from CD4^+^ T_H_2 cells and innate lymphoid cells, eosinophilic airway inflammation, and increased circulating IgE levels.^3^ However, non-T2 cytokines have also been implicated in asthma pathogenesis, including IL-17A,^4^, ^5^ IL-6,^6^ and IFN-γ.^7^, ^8^, ^9^ IFN-γ and IFN-γ–inducible type 1 (T1) immune gene expression is increased in the airways of a proportion of patients with severe asthma,^7^, ^8^, ^9^, ^10^ and IFN-γ drives corticosteroid-refractory airway hyperresponsiveness (AHR) in a murine severe asthma model.^9^ Importantly, patients with T1-high asthma coexpress T2 gene signatures to varying extents,^8^ suggesting that interplay between distinct T_H_ cell phenotypes might contribute to asthma pathology.

The anti-inflammatory cytokine IL-10 is a key regulator of the balance between T_H_ subsets during immune responses.^11^ IL-10 was originally described as a T_H_2-derived cytokine with the potential to suppress T_H_1 function by acting on antigen-presenting cells (APCs).^12^, ^13^, ^14^, ^15^ However, IL-10 can also suppress T2 and IL-17–dependent type 17 (T17) immune responses and be produced by T_H_1, T_H_2, and T_H_17 effector T (Teff) cells, as well as forkhead box P3 (FoxP3)^−^ and FoxP3^+^ regulatory T (Treg) cells, B cells, and several myeloid cell subsets.^11^, ^16^, ^17^ Therefore IL-10 can act through diverse mechanisms to limit pulmonary immunopathology in different contexts.^18^

T_H_ cell–derived IL-10 is a key mediator of induced tolerance to allergens^19^, ^20^ and resolution of allergic inflammation.^21^ Conversely, impaired IL-10 production by T_H_ cells has been reported in patients with severe asthma,^22^, ^23^ and genetic variants affecting IL-10 expression have been associated with asthma incidence and severity.^24^, ^25^ However, despite these associations, the mechanisms by which IL-10 regulates diverse immunologic phenotypes of asthma are incompletely understood.^18^

Mechanistic studies in asthma frequently use murine models of allergic airway disease (AAD) typically involving peripheral sensitization to a model allergen followed by airway allergen challenge.^26^ These protocols bypass the APC network of the lungs, which is essential for sensitization to inhaled allergens,^27^ and generate a highly polarized T2 response that does not reflect the heterogeneous immunologic phenotypes observed in human asthma.^1^, ^8^, ^26^ In contrast, sensitization through repeated allergen inhalation drives more complex immune responses, including T1 and T17 cytokines, in a manner dependent on activation of pulmonary innate immunity by endogenous or exogenous adjuvants, such as LPS^28^, ^29^, ^30^ or the bacterial messenger cyclic-di-GMP.^9^

To date, mechanistic studies of IL-10 function in patients with AAD have largely focused on T2-high peripheral sensitization/airway challenge models and shown IL-10 to limit T_H_2 cell survival, eosinophilic inflammation, and AHR.^31^, ^32^, ^33^ However, studies of IL-10 regulation of non-T2 immunity in patients with AAD are lacking.

In this work we showed repeated inhalation of the common aeroallergen house dust mite (HDM) to induce a mixed T2/T17 immune response and elicit IL-10 production from Teff cells. T cell–derived IL-10 was shown to suppress a pathogenic IFN-γ response to HDM by signaling to myeloid cells, limiting damage to the airway epithelium. These findings provide new insight into the context-specific nature of IL-10 function and regulation of non-T2 immunity in asthmatic patients.

## Methods

Detailed methods, including additional experimental procedures, are presented in the Methods section in this article's Online Repository at www.jacionline.org.

### Experimental animals

All animal work was performed in accordance with the Animals (Scientific Procedures) Act 1986 at Imperial College London. Female C57BL/6J mice were purchased from Charles River Laboratories (Margate, United Kingdom). 10BiT IL-10 reporter mice,^34^ *Il10*^fl/fl^ mice^35^ crossed to CD4-Cre,^36^ and *Il10ra*^fl/fl^ mice^37^ crossed to CD11c-Cre mice were obtained from the Francis Crick Institute (London, United Kingdom) and maintained at Charles River Laboratories. Littermate control mice and comparable proportions of male and female mice were used for all transgenic experiments. No sex-dependent differences were observed in experiments.

### Induction of AAD and cytokine blockade

Mice were anesthetized with isoflurane and administered 25 μg (total protein) of HDM (Citeq Biologics, Groningen, The Netherlands) in 25 μL of PBS (Thermo Fisher Scientific, Waltham, Mass) intranasally 5 times per week for 3 weeks. Control mice received 25 μL of PBS. HDM endotoxin content was 1.1 × 10^3^ EU/mg. In IL-10 receptor α (IL-10Rα) blockade experiments 50 μg/50 μL of 1B1.3a (αIL-10R) or RTK2071 rat IgG_1_ isotype UltraLEAF antibodies (BioLegend, San Diego, Calif) were administered intranasally 24 hours before the first allergen challenge and twice weekly thereafter. In IFN-γ blocking experiments 250 μg/250 μL of XMG1.2 (αIFN-γ) or HPRN rat IgG_1_ isotype *InVivo*Mab antibodies (Bio X Cell, West Lebanon, NH) were injected intraperitoneally 4 times on alternating days over the final 8 days of the HDM protocol. Allergen treatments were initiated at 6 to 9 weeks of age, and analysis was performed 24 hours after the final challenge.

### Measurement of airway function

Lung resistance was measured in response to increasing concentrations of methacholine (Sigma-Aldrich, St Louis, Mo), as described previously,^38^ by using the flexiVent apparatus and software (SCIREQ, Montreal, Quebec, Canada). Resistance was calculated by using flexiVent software with the following equation:

Pressure=(Resistance×Flow)+(Elastance×Volume)+Fitting constant.

### Epithelial disruption scoring

A previously published scoring strategy^39^ was modified for application to AAD. Hematoxylin and eosin–stained sections were scored by a blinded investigator and independently verified by a second blinded investigator. All complete airways (minimum of 5) on a section were scored on a 0- to 8-point scale, and mean scores were reported. Scoring was as follows: 1, minor ruffling of up to 50% of the airway epithelium; 2, ruffling of 50% or more of the airway epithelium; +1/+2 for severe morphological change (hypertrophy or vacuolation of cells) in up to 50% or 50% or more of the airway; and an additional +1 to airways with scores of 4 with severe loss of epithelial uniformity. Additional scores of +1, +2, or +3 were given for shedding of 1% to 25%, 25% to 50%, or greater than 50%, respectively, of epithelial cells.

### Statistical analysis

Analyses were performed with Prism software (version 7; GraphPad, La Jolla, Calif). Mann-Whitney *U* tests or Kruskal-Wallis tests with Dunn *post hoc* tests were used for single and multiple comparisons, respectively.

## Results

### CD4^+^ Teff cells are a major IL-10–producing population after repeated allergen inhalation

**Fig 1:**
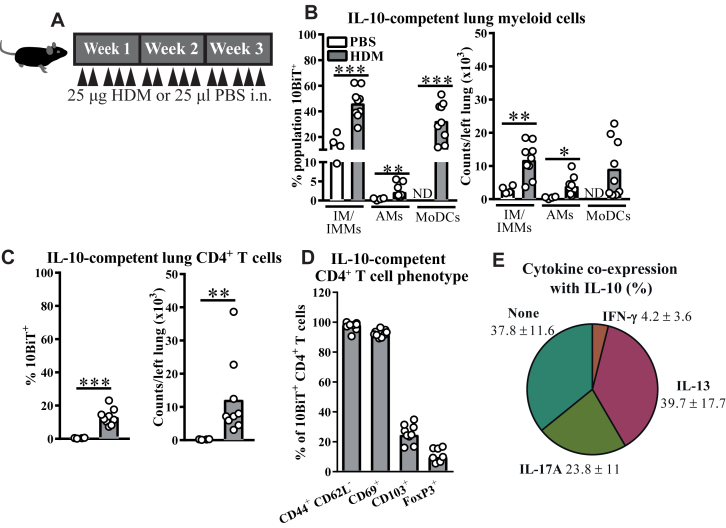
Effector CD4^+^ T cells are major IL-10 producers after repeated HDM inhalation. **A,** Experimental scheme. *i. n.*, Intranasal. **B-E,** Flow cytometric data. Fig 1, *B* and *C*, Percentages and absolute numbers of myeloid cells and CD4^+^ T cells in lung tissue expressing the 10BiT^+^ IL-10 reporter. Fig 1, *D*, Percentage of 10BiT^+^ CD4^+^ T cells from lungs of HDM-treated mice expressing the indicated markers. Fig 1, *E*, Percentages (median ± interquartile range) of IL-10^+^ CD4^+^ cells in the lungs of HDM-treated mice coexpressing the indicated cytokines after PMA and ionomycin stimulation. Data in Fig 1, *E*, represent 1 of 2 experiments (n = 6). Other data are pooled from 2 experiments (n = 4-9 per group). Data are shown as medians and individual replicates. *P < .05, **P < .01, and ***P < .001. *ND*, Not detected (insufficient moDC events).

**Fig E1:**
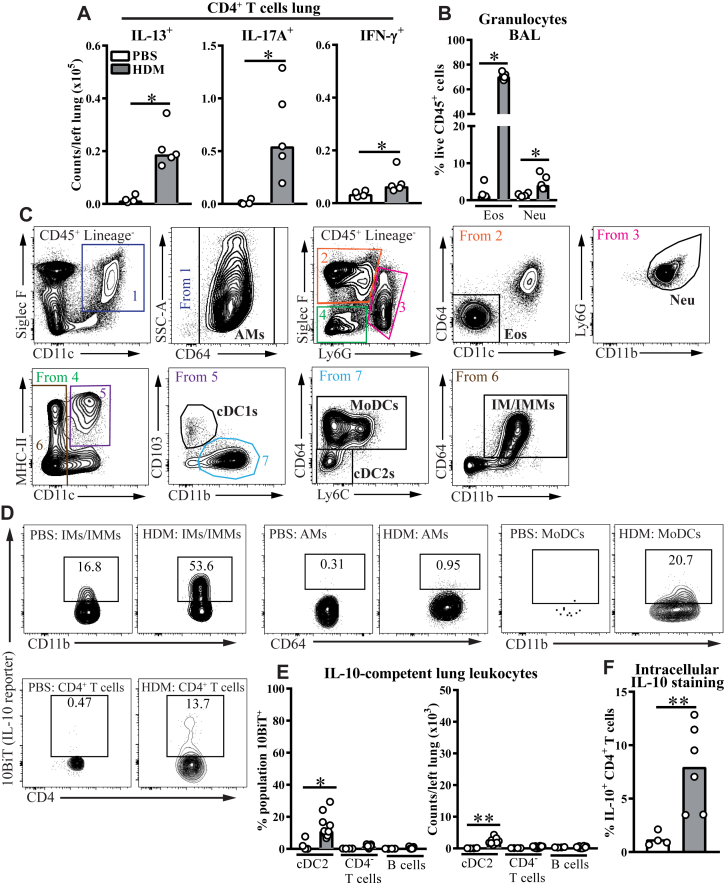
Cellular phenotype and IL-10 production in HDM inhalation mouse asthma model. Flow cytometric data are shown. **A,** Numbers of CD4^+^ T cells with the indicated cytokine expression phenotype in lung tissue of C57BL/6 mice. **B,** Siglec-F–high CD64^−^ eosinophils *(Eos)* and Ly6G-high CD11b-high neutrophils *(Neu)* as percentages of total CD45^+^ leukocytes in BAL fluid of C57BL/6 mice. **C,** Hierarchical strategy for gating mouse myeloid cells beginning with live, singlet, CD45^+^, lymphocyte lineage (CD90.2, CD19, NKp46)–negative cells. Representative plots from lungs of HDM-treated C57BL/6 mice are shown. **D,** Representative plots showing 10BiT reporter expression in the indicated populations. **E,** Quantification of percentages and absolute numbers of the indicated cell populations expressing the 10BiT reporter. **F,** Percentage of lung CD4^+^ T cells from C57BL/6 mice with intracellular IL-10 staining after PMA and ionomycin stimulation. Data shown are medians of displayed values. Data in Fig E1, *A*, *B*, and *F*, are from 1 of 2 experiments (n = 3-6 per group) showing comparable results. Data in Fig E1, *E*, are pooled from 2 experiments (n = 4-9 per group). *P < .05 and **P < .01.

To facilitate the study of IL-10 regulation of non-T2 immunity in asthmatic patients, we first established a complex T_H_ phenotype mouse AAD model using repeated administration of intranasal HDM for 3 weeks (Fig 1, *A*). This protocol elicited substantial accumulation of pulmonary IL-13^+^ and IL-17A^+^ T_H_ cells, with more IL-17A^+^ than IL-13^+^ cells and a modest increase in numbers of T_H_ cells producing IFN-γ (see Fig E1, *A*, in this article's Online Repository at www.jacionline.org). This protocol induced AHR (data not shown) and eosinophilic airway inflammation with minor neutrophilia (see Fig E1, *B*). Cells capable of IL-10 production (IL-10–competent cells) were identified in this model by using surface CD90.1 staining in 10BiT IL-10 reporter mice,^34^ first modifying a published myeloid cell flow cytometry strategy^40^ to distinguish airway macrophages (AMs), granulocytes, dendritic cell (DC) subsets, and a mixed population of interstitial macrophages (IMs) and inflammatory monocytes and macrophages (IMMs; see Fig E1, *C*). Consistent with published findings,^41^, ^42^ a population of IL-10–competent IMs/IMMs, likely resident IMs, was present in lungs of control (PBS-treated) mice (Fig 1, *B-E*), which expanded during HDM-driven AAD (Fig 1, *B*, and see Fig E1, *D*). In contrast, IL-10 reporter signal was largely absent from AMs at steady state and minimally increased during AAD, either in postlavage lung tissue (Fig 1, *B*, and see Fig E1, *D*) or in bronchoalveolar lavage (BAL) fluid (data not shown). Ten percent to 50% of HDM-elicited CD64^+^Ly6C^variable^ monocyte-derived dendritic cells (moDCs) were IL-10 competent, a substantial population in terms of absolute numbers, whereas IL-10–competent type 2 conventional dendritic cells (cDC2s) were less abundant in lung tissue (Fig 1, *B*, and see Fig E1, *D* and *E*). These results indicate that resident IMs and recruited monocyte-derived cells are the major myeloid cell sources of IL-10 after repeated HDM inhalation.

Among lymphoid cells, minimal IL-10 signal was detected in CD4^−^ T cells (CD45^+^CD3^+^) or B cells (CD45^+^CD3^−^CD19^+^) in lungs (see Fig E1, *E*) or draining mediastinal lymph nodes (mLNs) of 10BiT mice (data not shown). However, CD4^+^ T cells emerged as the major pulmonary IL-10–producing lymphocyte population during established allergic inflammation (Fig 1, *C*, and see Fig E1, *D*). Nearly all IL-10–competent CD4^+^ T cells had a CD44^hi^CD62L^−^CD69^+^ effector memory T-cell phenotype, with a minority expressing the tissue residency marker CD103 or the hallmark Treg transcription factor FoxP3 (Fig 1, *D*). *Ex vivo* phorbol 12-myristate 13-acetate (PMA) and ionomycin stimulation and intracellular cytokine staining confirmed around 5% to 15% of lung CD4^+^ T cells to be IL-10 producers (see Fig E1, *F*) and also showed more than half of these cells to coexpress either of the predominant HDM-elicited effector cytokines, IL-13 or IL-17A (Fig 1, *E*). Thus HDM inhalation largely drives IL-10 production from Teff cells rather than a discrete Treg cell population.

### T cell–derived IL-10 limits IFN-γ and IL-17A responses to inhaled HDM

**Fig 2:**
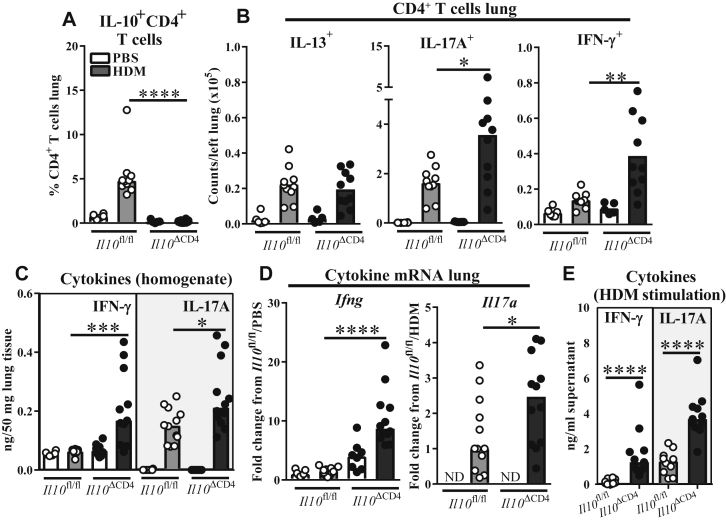
Lack of T cell–derived IL-10 augments pulmonary IFN-γ and IL-17A responses to HDM **A** and **B,** Flow cytometric data. Fig 2, *A*, Percentage of lung CD4^+^ T cells positive for IL-10 in *Il10*^ΔCD4^ knockout mice and control mice. Fig 2, *B*, Numbers of CD4^+^ T cells in lung tissue expressing the indicated cytokines. **C,** Cytokine concentrations in homogenized lung tissue. **D,** Fold changes (from the indicated control group) in cytokine gene expression in homogenized lung tissue. **E,** IFN-γ and IL-17A concentrations in supernatants of lung cell suspensions from HDM-treated mice stimulated with HDM for 4 days. Data were pooled from 2 to 3 experiments and shown as medians and individual replicates (n = 7-12 per group). *P < .05, **P < .01, ***P < .001, and ****P < .0001. *ND*, Not detected.

**Fig E2:**
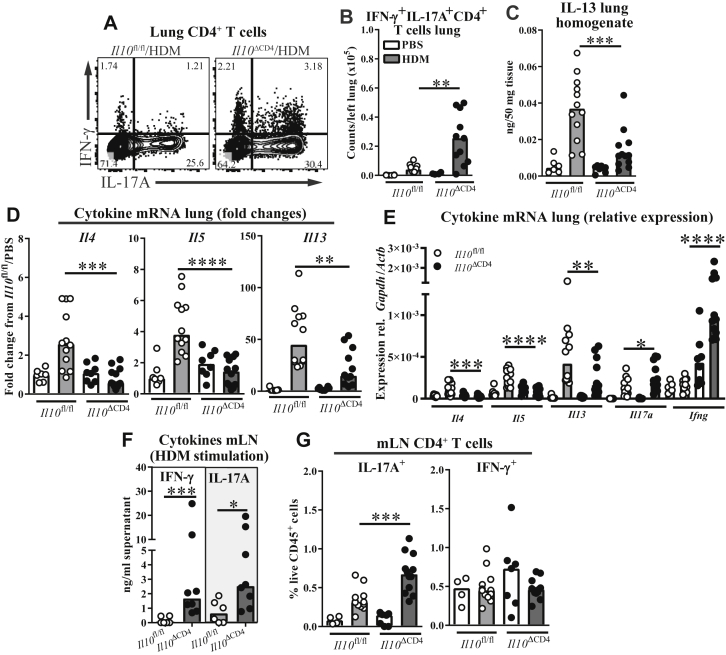
T_H_ cell cytokines in HDM-treated T cell–specific IL-10 knockout mice. **A,** Representative flow cytometric plots showing intracellular cytokine staining in lung CD4^+^ T cells. **B,** Quantification of numbers of double-positive cells in Fig E2, *A*. **C,** IL-13 protein concentration in lung homogenate. **D,** Fold changes in cytokine mRNA expression in homogenized lung tissue. **E,** 2^−ΔCT^ relative expression values of the indicated cytokines in lung tissue. **F,** IFN-γ and IL-17A concentrations in supernatants of mLN cell suspensions from HDM-treated mice stimulated with HDM for 4 days. **G,** IL-17A^+^ and IFN-γ^+^ CD4^+^ T cells as percentages of total CD45^+^ leukocytes in mLNs. Data are pooled from 2 experiments and shown as medians and individual replicates (n = 4-12 per group). *P < .05, **P < .01, ***P < .001, and ****P < .0001.

Given the proven importance of IL-10 in regulating T_H_2 responses to allergen *in vivo*,^19^, ^20^, ^21^, ^31^, ^32^, ^33^ we next asked whether the T cell–derived IL-10 elicited by means of HDM inhalation influenced the balance of T_H_ subsets in this model using CD4 conditional IL-10 knockout (*Il10*^ΔCD4^) mice^35^ and *ex vivo* PMA and ionomycin stimulation and intracellular cytokine staining of T_H_ cells. As expected, HDM-elicited IL-10^+^ T_H_ cells were completely ablated in *Il10*^ΔCD4^ mice (Fig 2, *A*). However, in contrast to previous reports of IL-10 signaling disruption in T2-high AAD models,^31^, ^32^ pulmonary IL-13^+^ T_H_ cell numbers were not increased in *Il10*^ΔCD4^ mice relative to those in control mice after repeated HDM inhalation. Instead, IL-17A^+^ and IFN-γ^+^ T_H_ cells were more abundant in lung tissue, including a double-positive population that was much less abundant in IL-10–replete mice (Fig 2, *B*, and see Fig E2, *A* and *B*, in this article's Online Repository at www.jacionline.org). Accordingly, levels of IFN-γ and IL-17A protein and mRNA were increased in lungs of HDM-treated *Il10*^ΔCD4^ mice and in supernatants of lung cell suspensions restimulated with HDM (Fig 2, *C-E*), suggesting that increased cytokine levels *in vivo* were attributable to allergen-specific T cells. In contrast, IL-13 protein concentrations were reduced in lungs of HDM-treated *Il10*^ΔCD4^ mice (see Fig E2, *C*), as was expression of the T2 cytokine genes *Il4*, *Il5*, and *Il13* (see Fig E2, *D* and *E*). Alignment of relative T_H_ cytokine expression (2^Δ-Ct^) values in lung tissue supported a skewing effect in knockout mice, with a reduction in the relatively high T2 cytokine expression observed in HDM-treated *Il10*^ΔCD4^ controls accompanied by increased *Il17a* and *Ifng* levels (see Fig E2, *E*).

T_H_ responses were also examined in mLNs to determine whether changes were restricted to lung tissue. Production of both IFN-γ and IL-17A production was increased in *Il10*^ΔCD4^ mice after HDM restimulation of mLN cells, but only IL-17A^+^ and not IFN-γ^+^ T_H_ cells were overrepresented in mLNs of knockout mice when analyzed directly *ex vivo* (see Fig E2, *F* and *G*). Thus T cell–derived IL-10 is required to restrain IFN-γ and IL-17A responses to inhaled HDM, with enhancement of the IFN-γ response being most pronounced at the site of allergen exposure in the lungs.

### Absence of T cell–derived IL-10 results in an eosinophil-low AAD phenotype with more severe epithelial pathology

**Fig 3:**
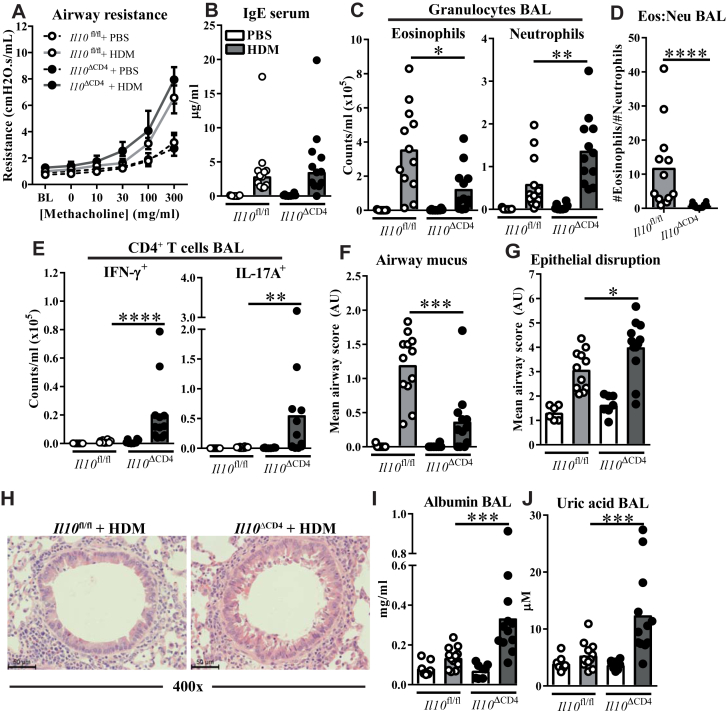
HDM drives eosinophil-low AAD with worsened airway pathology in mice lacking T cell–derived IL-10. **A,** Airway resistance to increasing doses of methacholine, as measured by using flexiVent. *BL*, Baseline. **B,** Total IgE concentrations in serum. **C-E,** Flow cytometric data. Fig 3, *C*, Total numbers of eosinophils and neutrophils in BAL fluid. Fig 3, *D*, Ratio of eosinophils to neutrophils in BAL fluid. Fig 3, *E*, Numbers of IFN-γ^+^ and IL-17A^+^ CD4^+^ T cells in BAL fluid. **F** and **G,** Semiquantitative scores of PAS mucus staining (Fig 3, *F*) and lung airway epithelial disruption (Fig 3, *G*). **H,** Representative airway images from hematoxylin and eosin–stained lung sections quantified in Fig 3, *G*. **I** and **J,** Concentrations of albumin and uric acid in BAL fluid. Data are pooled from 2 experiments. Fig 3, *A*, shows medians ± interquartile ranges; other panels show medians and individual replicates (n = 7-12 per group). *P < .05; **P < .01, ***P < .001, and ****P < .0001.

**Fig E3:**
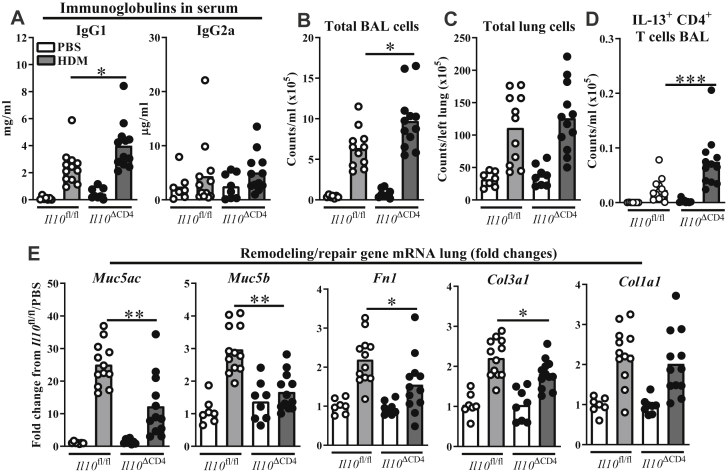
AAD parameters in HDM-treated T cell–specific IL-10 knockout mice. **A,** Concentrations of total IgG_1_ and IgG_2a_ in serum. **B** and **C,** Total cell counts from BAL fluid (Fig E3, *B*) and digested lung tissue (Fig E3, *C*). **D,** Flow cytometric data showing IL-13^+^ CD4^+^ T-cell numbers in BAL fluid. **E,** Fold changes in mRNA expression of the indicated genes in homogenized lung tissue. Data are pooled from 2 experiments (n = 7-12 per group). *P < .05, **P < .01, and ***P < .001.

We next determined whether the skewed T_H_ response to HDM in *Il10*^ΔCD4^ mice was associated with changes to the hallmark pathophysiologic features of allergic asthma. HDM-treated *Il10*^ΔCD4^ mice had comparable lung resistance in response to inhaled methacholine and serum IgE levels to littermate control mice (Fig 3, *A* and *B*), indicating that the heightened IFN-γ/IL-17A responses in these mice did not impair allergic sensitization or prevent AHR. Serum IgG_1_ concentrations were slightly increased in *Il10*^ΔCD4^ mice, whereas little to no IgG_2a_ induction was observed in either group (see Fig E3, *A*, in this article's Online Repository at www.jacionline.org).

HDM drove cellular inflammation in the airways and lung tissue of both genotypes, with significantly more BAL fluid cells in *Il10*^ΔCD4^ mice compared with *Il10*^fl/fl^ control mice (see Fig E3, *B* and *C*). Despite this overall increase, BAL fluid eosinophilia was significantly reduced in *Il10*^ΔCD4^ mice accompanied by increased neutrophil numbers, together skewing the eosinophil/neutrophil ratio toward an eosinophil-low phenotype (Fig 3, *C* and *D*). Numbers of CD4^+^ T cells expressing IFN-γ and IL-17A were also increased in BAL fluid of *Il10*^ΔCD4^ mice and were largely absent in control mice (Fig 3, *E*). IL-13^+^ T_H_ cells were more abundant in BAL fluid of knockout mice, although at lower numbers than IFN-γ^+^ or IL-17A^+^ cells (see Fig E3, *D*). Airway mucus was increased in both groups after HDM exposure but was less substantial in *Il10*^ΔCD4^ mice (Fig 3, *F*), which was congruent with reduced expression of the mucin genes *Muc5ac* and *Muc5b* (Fig E3, *E*). In addition, expression of genes indicative of early airway repair/remodeling responses, fibronectin *(Fn1)* and type III collagen *(Col3a1)*, but not type I collagen *(Col1a1)*, was also dampened in HDM-treated *Il10*^ΔCD4^ mice (see Fig E3, *E*).

These changes prompted us to further examine the airway epithelium for imbalances in damage and repair by using composite scoring of disruption of the epithelial layer. More severe epithelial disruption was observed in HDM-treated *Il10*^ΔCD4^ mice than control mice (Fig 3, *G* and *H*), along with increased airway levels of albumin (Fig 3, *I*), which is suggestive of leakage of serum proteins across the epithelial barrier, and uric acid, which is associated with epithelial damage and stress (Fig 3, *J*).^43^, ^44^ Although neutrophils were more abundant in the airways of *Il10*^ΔCD4^ mice (Fig 3, *C*) and can contribute to airway epithelial damage in certain contexts,^45^ no evidence of infiltration of damaged epithelium was observed in lung sections (data not shown). Collectively, our data show that cell-derived IL-10 restricts atypical immunity to inhaled HDM and limits airway epithelial pathology.

### Local pulmonary IL-10 signaling is required to suppress the IFN-γ response to HDM

**Fig 4:**
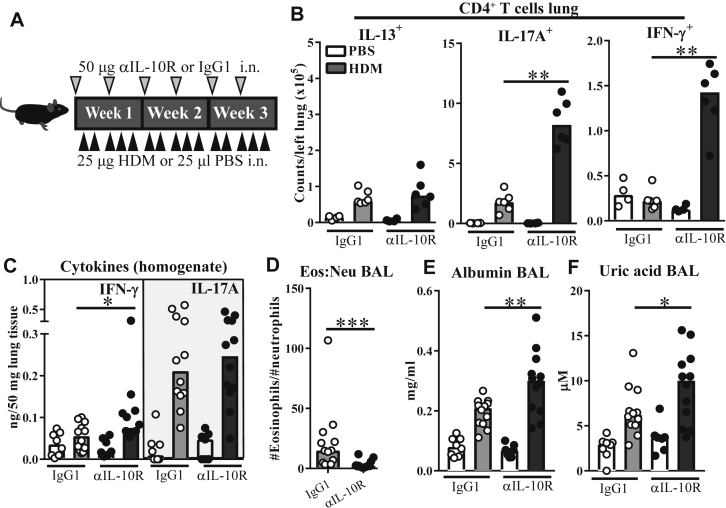
Blockade of pulmonary IL-10R signaling is sufficient to drive an IFN-γ–high, eosinophil-low response to HDM. **A,** Experimental scheme. *i. n.*, Intranasal. **B,** Flow cytometric data showing numbers of CD4^+^ T cells with the indicated cytokine phenotype in lung tissue. **C,** Concentrations of IFN-γ and IL-17A in homogenized lung tissue. **D,** Ratio of eosinophils *(Eos)* to neutrophils *(Neu)* in BAL fluid of HDM-treated mice, as determined by using flow cytometry. **E** and **F,** Concentrations of albumin and uric acid in BAL fluid. Data in Fig 4, *B*, are from 1 of 2 experiments showing comparable results (n = 3-6 per group). Other data are pooled from 2 experiments, (n = 7-12 per group). Data are shown as medians and individual replicates. *P < .05, **P < .01, and ***P < .001.

**Fig E4:**
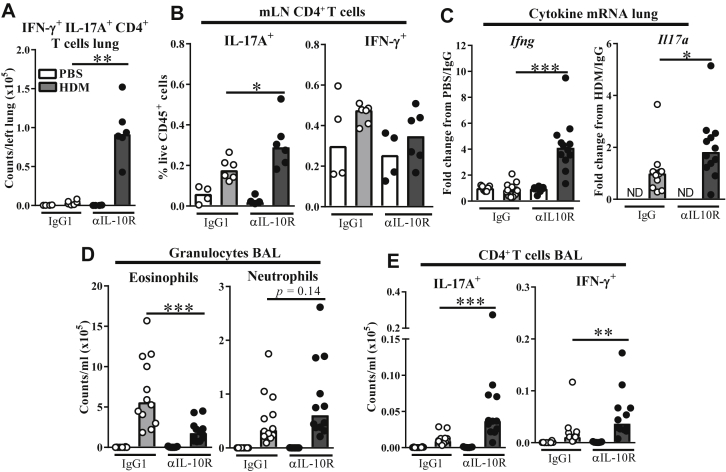
Additional effects of local IL-10R blockade on the immune response to HDM. **A** and **B,** Flow cytometric data. Fig E4, *A*, Numbers of IL-17A/IFN-γ double-positive CD4^+^ T cells in lung tissue. Fig E4, *B*, IL-17A^+^ and IFN-γ CD4^+^ T cells as percentages of total CD45^+^ leukocytes in mLNs. **C,** Fold changes (from the indicated group) of *Ifng* and *Il17a* mRNA expression in homogenized lung tissue. **D** and **E,** Flow cytometric data showing numbers of eosinophils, neutrophils, and IL-17A^+^ and IFN-γ CD4^+^ T cells in BAL fluid. Data in Fig E4, *A* and *B*, are from 1 of 2 experiments showing comparable results (n = 3-6 per group). Other data are pooled from 2 experiments (n = 7-12 per group). Data are shown as medians and individual replicates. *P < .05, **P < .01, and ***P < .001. *ND*, Not detected.

We next investigated whether the effects of T cell–specific IL-10 knockout could be recapitulated by panblockade of IL-10 signaling in cells residing in or migrating from lung tissue. Low-dose blocking antibody to the unique IL-10Rα subunit of the IL-10 receptor (αIL-10R) was administered intranasally throughout the HDM protocol to preferentially block pulmonary IL-10 signaling (Fig 4, *A*). Local IL-10R blockade caused remarkably similar effects to T cell–specific IL-10 knockout on pulmonary T_H_ cell numbers, increasing IL-17A^+^ and IFN-γ^+^ T_H_ cell numbers, including double-positive cells (Fig 4, *B*, and see Fig E4, *A*, in this article's Online Repository at www.jacionline.org). IL-10Rα blockade also increased IL-17A^+^, but not IFN-γ^+^, T_H_ cell abundance in mLNs (see Fig E4, *B*), as observed in *Il10*^ΔCD4^ mice (see Fig E2, *G*). Accordingly, lung IFN-γ mRNA and protein levels were increased after IL-10R blockade, whereas a modest increase in IL-17A mRNA, but not protein, expression was observed (Fig 4, *C*, and see Fig E4, *C*), suggesting that local IL-10 signaling was more influential on the IFN-γ than IL-17A response to HDM. IL-10R blockade also partially recapitulated the eosinophil-low airway inflammation phenotype observed in *Il10*^ΔCD4^ mice (Fig 4, *D*, and see Fig E4, *D*) and resulted in increased numbers of IFN-γ^+^ and IL-17A^+^ T_H_ cells in BAL fluid (see Fig E4, *D*). This altered inflammatory phenotype was accompanied by increased BAL fluid albumin and uric acid concentrations (Fig 4, *E* and *F*), which is suggestive of more severe airway damage. This parity between the results of pan–IL-10Rα blockade in the lungs and T cell–specific IL-10 knockout suggests that T cells are the functionally dominant IL-10 source acting locally in lung tissue to limit atypical immunologic and pathologic responses to inhaled HDM.

### Pulmonary myeloid cell dysregulation in the absence of T cell–derived IL-10

**Fig 5:**
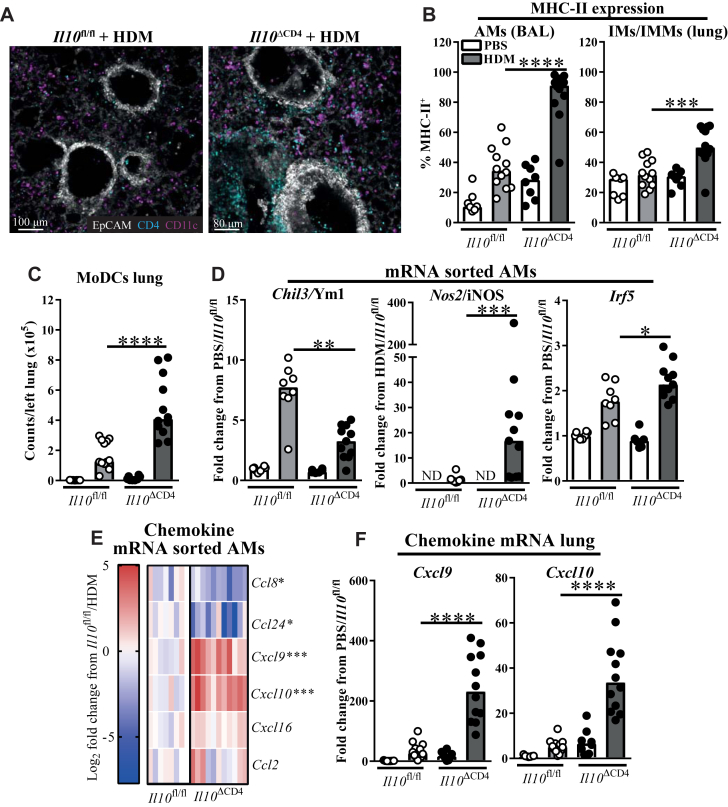
Pulmonary myeloid cell dysfunction during AAD in the absence of T cell–derived IL-10. **A,** Representative images of precision-cut lung slices from HDM-treated mice showing colocalization of CD4^+^ T cells and CD11c^+^ myeloid cells. **B** and **C,** Flow cytometric data. Fig 5, *B*, Percentage of MHC class II^+^ cells among AMs and IMs/IMMs in BAL fluid and lung tissue, respectively. Fig 5, *C*, Numbers of moDCs in lung tissue. **D,** Fold changes (from the indicated experimental group) of *Chil3*, *Nos2*, and *Irf5* mRNA expression in AMs sorted by means of fluorescence-activated cell sorting. **E,** Heat map showing altered chemokine gene expression in AMs sorted from HDM-treated *Il10*^ΔCD4^ mice. **F,** Fold changes in *Cxcl9* and *Cxcl10* mRNA expression in homogenized lung tissue. Fig 5, *E*, shows log_2_ fold changes from HDM-treated *Il10*^fl/fl^ control mice pooled from 3 experiments (n = 8-11 per group). Remaining data are pooled from 2 to 3 experiments and show medians and individual replicates (n = 6-12 per group). *P < .05, **P < .01, ***P < .001, and ****P < .0001. *ND*, Not detected.

**Fig E5:**
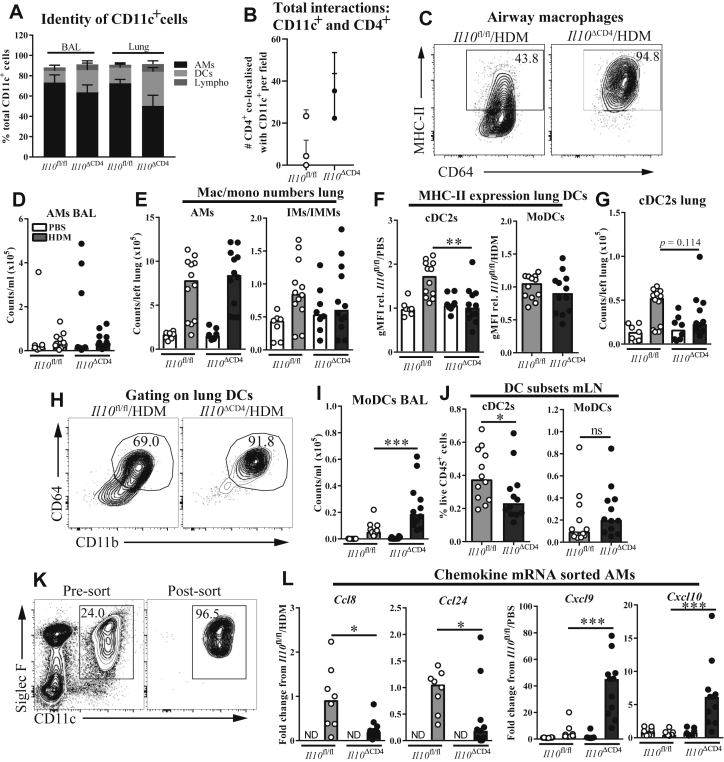
Myeloid cells in HDM-treated T cell–specific IL-10 knockout mice. **A,** Percentages of total live CD11c^+^ cells with the AM (lineage-negative CD11c^+^ Siglec-F^+^ CD64^+^), DC (lineage/Ly6G/Siglec-F^−^ CD11c^+^ MHC class II^+^ CD11b^+^ or CD103^+^), or lymphocyte (*Lympho*; lineage-positive forward scatter/side scatter low) phenotype, as determined by using flow cytometry. **B,** Numbers of CD4^+^ cells within 10 μm of a CD11c^+^ cell in precision-cut lung slices. **C-K,** Flow cytometric data. Fig E5, *C*, Representative plots showing MHC class II expression by AMs. Fig E5, *D* and *E*, Numbers of AMs in BAL fluid and lung tissue and IMs/IMMs in lung tissue. Fig E5, *F*, Fold differences (from indicated group) in geometric mean fluorescence intensity of MHC class II staining on cDC2s and moDCs in lung tissue. Fig E5, *G*, Total cDC2 numbers in lung tissue. Fig E5, *H*, Plots showing moDCs (gated) among total DCs in lung tissue. Fig E5, *I*, Total moDC numbers in BAL fluid. Fig E5, *J*, cDC2s and moDCs as percentages of total CD45^+^ leukocytes in mLNs. Fig E5, *K*, Representative plots showing total cells from BAL fluid before and after AM sorting. **L,** Fold changes in mRNA expression of chemokines summarized in Fig 5, *K*, in AMs sorted by means of fluorescence-activated cell sorting. Fig E5, *A*, shows median percentages ± interquartile ranges pooled from 2 experiments (n = 7-12 per group). Points in Fig 5, *B*, show means ± SDs of 3 equally sized fields from each of 2 to 3 mice per group. All other data show medians and individual replicates and are pooled from 2 to 3 experiments (n = 7-12 per group). *P < .05, **P < .01, and ***P < .001. *ND*, Not detected; *ns*, not significant.

Because IL-10 can suppress T_H_1 cell responses by acting on APCs^14^, ^15^ and IFN-γ can show positive feedback to APCs to perpetuate T_H_1 immunity, we investigated whether cross-talk between lung macrophages, DCs, and T_H_ cells was dysregulated during the imbalance of IFN-γ and IL-10 observed in HDM-exposed *Il10*^ΔCD4^ mice. We first validated integrin CD11c as a specific marker of AMs, the major pulmonary macrophage subset, and DCs^40^ in the lung. AMs were confirmed to be the predominant CD11c^+^ population in BAL fluid and lung tissue after HDM treatment, followed by DCs (see Fig E5, *A*, in this article's Online Repository at www.jacionline.org), which were predominantly CD64^+^CD11b^+^CD103^−^ moDCs in both the *Il10*^fl/fl^ and *Il10*^ΔCD4^ groups. CD4^+^ T cells could be observed in close (≤10 μm) proximity to CD11c^+^ AMs/DCs in live *ex vivo* lung tissue of HDM-treated mice, and these interactions were more frequent in *Il10*^ΔCD4^ mice, which is consistent with the increased T-cell numbers seen in these animals (Fig 5, *A*, and see Fig E5, *B*), suggesting that local myeloid–T-cell cross-talk might be altered in the absence of IL-10.

Consistent with macrophage dysregulation, expression of the activation marker MHC class II was increased on AMs and pulmonary CD11c^−^ IMs/IMMs in HDM-treated *Il10*^ΔCD4^ mice (Fig 5, *B*, and see Fig E5, *C*), despite numbers of these cells being unchanged from control mice (see Fig E5, *D* and *E*). MHC class II expression was not increased on cDC2s or moDCs, the critical DC subsets for HDM-driven immunity,^27^ from *Il10*^ΔCD4^ mice and was in fact decreased on cDC2s (see Fig E5, *F*). However, although pulmonary cDC2 numbers were comparable, moDCs were more abundant in lung tissue and BAL fluid of HDM-treated *Il10*^ΔCD4^ mice (Fig 5, *C*, and see Fig E5, *G-I*), suggesting that moDCs might locally promote HDM-driven inflammation in these mice, as previously proposed.^27^ Neither DC population was more abundant in mLNs of *Il10*^ΔCD4^ mice, with a slight decrease in the proportion of cDC2s (see Fig E5, *J*). These data suggest that pulmonary macrophages and moDCs are dysregulated in the absence of T cell–derived IL-10 and might contribute to the atypical immune response to HDM in these mice.

### Dysregulated AMs overproduce T_H_1 cell chemokines

We next sought to better understand pulmonary macrophage dysregulation in *Il10*^ΔCD4^ mice, focusing on AMs because of their superior numbers and decisive role in regulating immunity to HDM.^46^ AMs were sorted from BAL fluid of HDM-treated *Il10*^ΔCD4^ mice and control mice (see Fig E5, *K*), and gene expression was examined. In line with the altered cytokine environment, AMs from *Il10*^ΔCD4^ mice showed decreased expression of the IL-4/IL-13–induced gene *Chil3*/Ym1 and concomitant increases in the IFN-γ–inducible nitric oxide synthase *Nos2*/iNOS and *Irf5* (Fig 5, *D*), a transcriptional regulator^47^ of classical macrophage activation that dictates AM phenotype during AAD.^46^

We have previously found AMs to be major producers of the IL-4/IL-13–inducible chemokines CCL8 and CCL24 in models of AAD (Branchett et al, unpublished results),^48^ which contribute to T2 immunity.^49^, ^50^ Gene expression analysis in sorted AMs showed expression of these T2 chemokines to be decreased, whereas levels of the IFN-γ–inducible CXCR3 ligands *Cxcl9* and *Cxcl10*, which are associated with amplification of T1 immune responses,^51^ were increased with HDM treatment in AMs from *Il10*^ΔCD4^ mice (Fig 5, *E*, and see Fig E5, *L*). These CXCR3 ligands were also more highly expressed in lungs of *Il10*^ΔCD4^ mice (Fig 5, *F*). Collectively, these data suggest that dysregulated AMs contribute to feedforward amplification of T1 immunity in the absence of T cell–derived IL-10 through upregulation of IFN-γ–inducible CXCR3 ligands.

### IL-10 suppression of HDM-induced IFN-γ and epithelial pathology relies on direct signaling to CD11c^+^ cells

**Fig 6:**
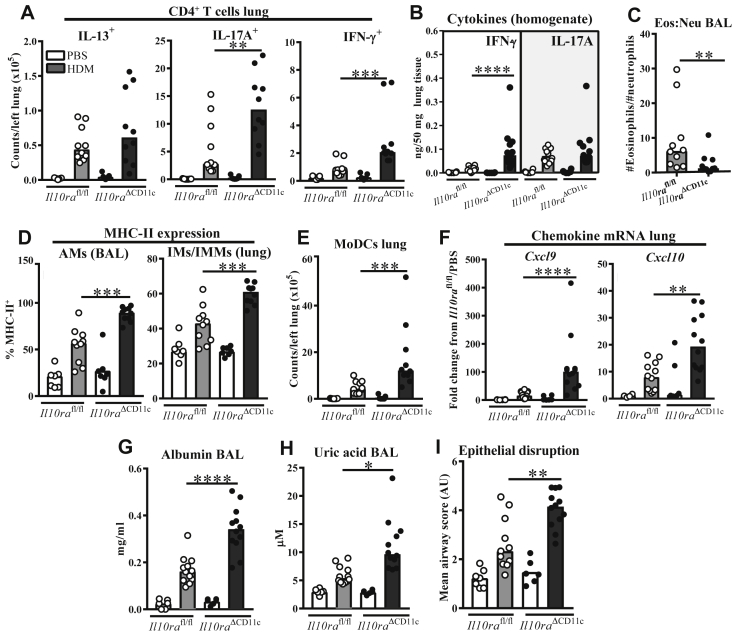
The IFN-γ response to HDM and epithelial damage are limited by IL-10 signaling to CD11c^+^ myeloid cells. **A,** Flow cytometric data showing numbers of CD4^+^ T cells in lung tissue with the indicated cytokine phenotype. **B,** Concentrations of IFN-γ and IL-17A in homogenized lung tissue. **C-E,** Flow cytometric data showing ratio of eosinophils *(Eos)* to neutrophils *(Neu)* in BAL fluid of HDM-treated mice (Fig 6, *C*), percentages of BAL fluid AMs and lung IMs/IMMs expressing MHC class II (Fig 6, *D*), and numbers of moDCs in lung tissue (Fig 6, *E*). **F,** Fold changes in *Cxcl9* and *Cxcl10* mRNA expression in homogenized lung tissue. **G** and **H,** Concentrations of albumin and uric acid in BAL fluid. **I,** Composite airway epithelial disruption scores of hematoxylin and eosin–stained lung sections. Data are pooled from 2 experiments and show medians and individual replicates (n = 6-12 per group). *P < .05, **P < .01, ***P < .001, and ****P < .0001.

**Fig E6:**
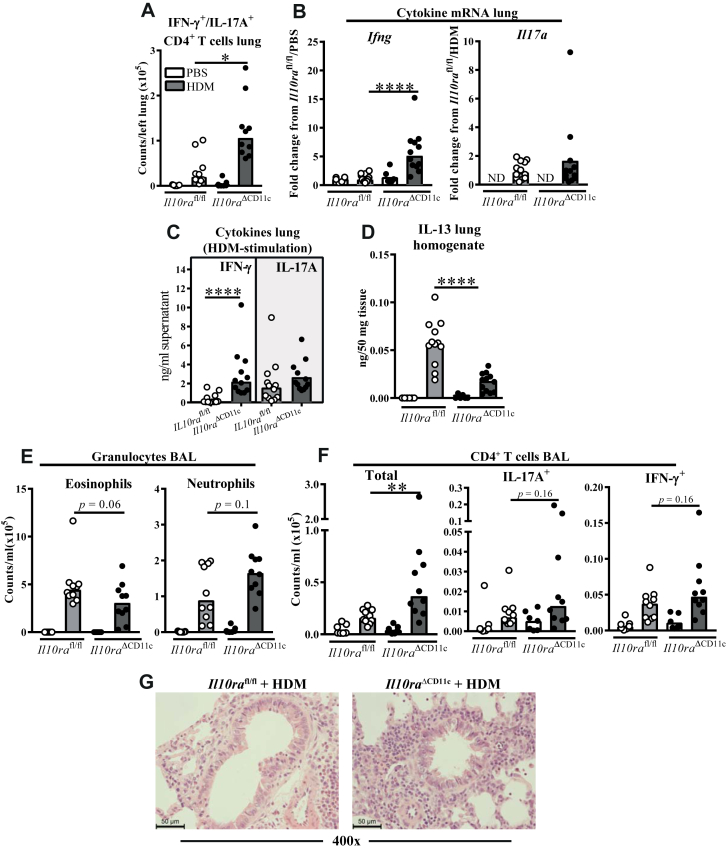
Immune response to HDM in *Il10ra*^ΔCD11c^ mice. **A,** IFN-γ/IL-17A double-positive CD4^+^ T cells, as determined by using flow cytometry. **B,** Fold changes in *Ifng* and *Il17a* mRNA expression in homogenized lung tissue. **C** and **D,** Concentrations of cytokines in supernatants of HDM-stimulated lung cell suspensions (Fig E6, *C*) and homogenized lung tissue (Fig E6, *D*). **E** and **F,** Flow cytometric data showing numbers of eosinophils and neutrophils (Fig E6, *E*) and IFN-γ^+^ and IL-17A^+^ CD4^+^ T cells (Fig E6, *F*) in BAL fluid. **G,** Representative images of hematoxylin and eosin–stained lung sections showing epithelial damage quantified in Fig 6. Data are pooled from 2 experiments and show medians and individual replicates (n = 4-13 per group). *P < .05, **P < .01, and ****P < .0001. *ND*, Not detected.

Having demonstrated dysregulation of CD11c^+^ myeloid cells during the aberrant IFN-γ response to HDM in the absence of T cell–derived IL-10, we next investigated whether IL-10 signals directly to these cells to suppress this phenotype. CD11c-Cre mice, which allow conditional gene deletion in AMs, lymphoid tissue DCs, and the majority of lung DCs,^52^, ^53^ were crossed to *Il10ra*^fl/fl^ mice^37^ to generate *Il10ra*^ΔCD11c^ conditional knockouts and exposed to HDM for 3 weeks. *Il10ra*^ΔCD11c^ mice had comparable increases in HDM-elicited IFN-γ^+^ and IL-17A^+^ pulmonary T_H_ cells with those observed in *Il10*^ΔCD4^ and local IL-10R blockade experiments (Fig 6, *A*, and see Fig E6, *A*, in this article's Online Repository at www.jacionline.org), along with significantly more IFN-γ (both protein and mRNA) in lung tissue (Fig 6, *B*, and see Fig E6, *B*) and supernatants of HDM-stimulated lung cells (see Fig E6, *C*). In contrast, IL-17A levels were unchanged in these assays (Fig 6, *B*, and see Fig E6, *B* and *C*), whereas IL-13 protein concentrations were lower in lungs of *Il10ra*^ΔCD11c^ mice (see Fig E6, *D*). These results indicate that, similarly to local IL-10R blockade (Fig 4), conditional IL-10R knockout more markedly enhanced the IFN-γ response to HDM than the IL-17A response, which was already substantial in mice with intact IL-10 signaling in our HDM model.

*Il10ra*^ΔCD11c^ mice also had a reduced airway eosinophil/neutrophil ratio (Fig 6, *C*, and see Fig E6, *E*) and increased numbers of total CD4^+^ T cells in BAL fluid, although numbers of IFN-γ^+^ and IL-17A^+^ cells in this compartment were not significantly altered (see Fig E6, *F*). HDM-treated *Il10ra*^ΔCD11c^ mice also displayed pulmonary APC dysregulation, which is apparent from increased MHC class II expression on AMs and IMs/IMMs and greater moDC numbers in lung tissue (Fig 6, *D* and *E*), along with increased pulmonary *Cxcl9* and *Cxcl10* levels (Fig 6, *F*), which is suggestive of increased CXCR3 ligand expression during the imbalance of IFN-γ and IL-10 signaling to myeloid cells. Importantly, the IFN-γ–high phenotype in *Il10ra*^ΔCD11c^ mice was accompanied by increased markers and histologic evidence of airway damage (Fig 6, *G-I*, and see Fig E6, *G*). Overall, the similarity of these results to those in *Il10*^ΔCD4^ mice suggests that T cell–derived IL-10 signals to CD11c^+^ myeloid cells, regulating T_H_1 cell–recruiting chemokines, HDM-elicited IFN-γ responses, and airway epithelial damage.

### IFN-γ drives pathogenic airway damage during AAD in the absence of T cell–derived IL-10

**Fig 7:**
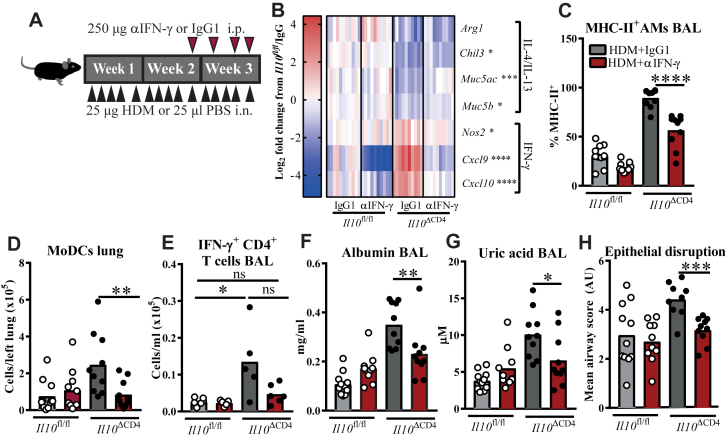
IFN-γ contributes to airway pathology in the absence of T cell–derived IL-10. **A,** Experimental scheme. *i. n.*, Intranasal; *i. p.*, intraperitoneal. **B,** Heat map showing changes in IL-4/IL-13 and IFN-γ signature genes in homogenized lung tissue. *Asterisks* refer to comparisons between αIFN-γ–and IgG-treated *Il10*^ΔCD4^ mice. **C-E,** Flow cytometric data showing percentages of BAL fluid AMs expressing MHC class II (Fig 7, *C*), numbers of moDCs in lung tissue (Fig 7, *D*), and IFN-γ^+^ CD4^+^ T cells in BAL fluid (Fig 7, *E*). **F** and **G,** Concentrations of albumin and uric acid in BAL fluid. **H,** Composite airway epithelial disruption scores of hematoxylin and eosin–stained lung sections. Fig 7, *B*, shows data from 2 experiments as log_2_ fold changes from the mean of their respective *Il10*^fl/fl^/IgG control group. Fig 7, *E*, shows 1 of 2 experiments with comparable trends (n = 5-6). Other panels show medians and individual replicates from 2 pooled experiments (n = 10-11 per group). *P < .05, **P < .01, ***P < .001, and ****P < .0001. *ns*, Non significant.

**Fig E7:**
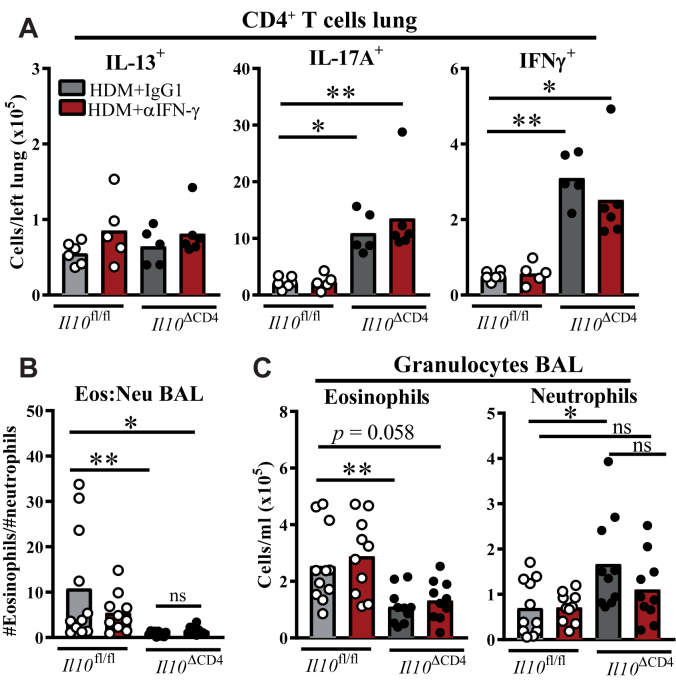
Therapeutic IFN-γ blockade partially corrects the inflammatory phenotype in *Il10*^ΔCD4^ mice. Flow cytometric data are shown. **A,** Numbers of CD4^+^ T cells with the indicated cytokine phenotype in lung tissue. **B,** Ratio of eosinophil *(Eos)* to neutrophil *(Neu)* numbers in BAL fluid. **C,** Absolute numbers of eosinophils and neutrophils in BAL fluid. Data in Fig E7, *A*, are representative of 2 experiments (n = 5-6 per group) showing comparable trends. Data in Fig E7, *B* and *C*, are pooled from 2 experiments (n = 10-11 per group). Data shown are medians and individual replicates. *P < .05 and **P < .01. *ns*, Nonsignificant.

Finally, anti–IFN-γ (αIFN-γ) was administered systemically to *Il10*^ΔCD4^ mice and control mice throughout the final week of HDM exposure to assess whether IFN-γ was responsible for the increased HDM-driven airway damage observed after disruption of the T_H_-CD11c^+^ myeloid cell IL-10 axis (Fig 7, *A*). Therapeutic αIFN-γ administration did not affect pulmonary cytokine-producing T_H_ cell numbers (see Fig E7, *A*, in this article's Online Repository at www.jacionline.org) but partially corrected the altered expression of IL-4/IL-13 and IFN-γ signature genes in lungs of *Il10*^ΔCD4^ mice, particularly the T_H_1 chemokines *Cxcl9* and *Cxcl10* (Fig 7, *B*), indicating successful suppression of IFN-γ signaling. Therapeutic αIFN-γ reduced the heightened MHC class II expression on AMs and lung moDC numbers in *Il10*^ΔCD4^ mice (Fig 7, *C* and *D*), which is consistent with these myeloid cell dysfunction phenotypes being IFN-γ dependent. Although therapeutic αIFN-γ did not restore eosinophilia in *Il10*^ΔCD4^ mice, it reduced numbers of neutrophils (see Fig E7, *B* and *C*) and IFN-γ^+^ CD4^+^ T cells (Fig 7, *E*) in BAL fluid to levels comparable with those in *Il10*^fl/fl^ control mice, suggesting that IFN-γ promotes airway inflammation in these mice. Importantly, airway albumin and uric acid levels were diminished by αIFN-γ treatment, which was congruent with an improved epithelial pathology score (Fig 7, *F-H*). These data confirm that high levels of IFN-γ arising during HDM-driven AAD in the absence of intact pulmonary IL-10 signaling contribute to airway pathology.

## Discussion

Pulmonary immunity must be tightly regulated by anti-inflammatory cytokines to minimize tissue damage that might compromise respiratory function.^18^ Asthma is thought to occur because of an imbalance between immune activation and regulation in the respiratory tract, prompting efforts to boost regulatory mechanisms by immunotherapy to treat allergic diseases.^54^ The cytokine IL-10 can regulate diverse T_H_ cell responses to limit immunopathology,^11^ including T2 immunity in murine models of asthma.^31^, ^32^ However, there is increasing evidence of heterogeneous asthma phenotypes in human subjects in whom dysfunctional T2 immunity is not the sole immunologic feature, and it is not known how such T2-low/non-T2 immunity is regulated in the setting of asthma.

Using complex mouse models of mucosal HDM sensitization and conditional gene deletion, we have examined the interactions between IL-10 and key proinflammatory cytokines in the allergic lung. Although IL-10 can be produced by several different cell types in the respiratory tract,^18^ we have shown IL-10–producing CD4 T cells, principally FoxP3^−^ Teff cells, to be elicited by repeated HDM exposure and that T cell–derived IL-10 signals to CD11c^+^ myeloid cells to regulate pathogenic IFN-γ responses to inhaled allergen. Parity between our results using specific knockout of T cell–derived IL-10 and global blockade of pulmonary IL-10 signaling suggest that CD4^+^ Teff cells are the functionally dominant IL-10–producing cells for limiting IFN-γ responses in the lungs in patients with HDM allergy. However, it remains possible that non-T2 responses to allergen can be regulated by IL-10–producing myeloid cells. Indeed, others have reported that adoptive transfer of IL-10–producing IMs can reduce neutrophilic T17 immune responses to HDM in IL-10 knockout mice.^55^ Our findings align with several mechanistic studies showing CD4^+^ T cells to be the functionally dominant IL-10 source in dampening protective or pathogenic immunity to respiratory tract infection^56^, ^57^, ^58^ and likely reflect negative feedback of Teff cell responses by intrinsic IL-10, restricting APC function to limit atypical and pathogenic IFN-γ responses to inhaled HDM.

Although knockout of T cell–derived IL-10 increased IFN-γ levels in the lung after HDM inhalation, it did not alter lung tissue IL-13^+^ T_H_ cell numbers and actually decreased pulmonary T2 cytokine levels and airway eosinophilia. This phenotype differs markedly from results in T2-high peripheral sensitization/airway challenge AAD models, in which disruption of endogenous IL-10 function augmented T2 immunity and AHR,^32^, ^33^ whereas lack of direct IL-10–signaling to T cells specifically enhanced T_H_2 cell survival and downstream allergic inflammation.^31^ This disparity with previous reports likely reflects engagement of distinct immunologic pathways by different allergens or sensitization routes. Previous studies have highlighted the importance of inhaled endotoxin for driving such differences because endotoxin can facilitate generation of mixed T2/T17 airway responses at lower concentrations^28^ and T1 responses at greater concentrations.^30^ Therefore we propose that T cell–derived IL-10 acts to limit T1 responses to the endogenous endotoxin present in HDM preparations, such that an IFN-γ response is unleashed in which IL-10 signaling is disrupted during repeated airway HDM exposure. Differences between the effects of IL-10 signaling disruption in our HDM inhalation experiments and the increased T_H_2 cell survival observed by Coomes et al^31^ in a peripheral sensitization HDM model might also result from the latter study specifically disrupting IL-10 signaling to T cells, whereas we instead targeted a T-cell–myeloid cell signaling axis.

Our data underscore the concept that the effects of a particular cytokine *in vivo* depend on its cellular source and cross-talk with other context-specific signals, which in turn depend on the nature of the inflammatory stimulus. Therefore it is important to evaluate cytokine function in diverse models of AAD, particularly those such as ours in which sensitization occurs through the physiologically relevant airway route in the absence of systemic adjuvant.

The effects of T cell–restricted IL-10 deletion on IFN-γ production could be recapitulated by panblockade of local pulmonary IL-10R signaling through the airways or deletion of IL-10Rα from CD11c^+^ AMs and DCs, suggesting that Teff cells signal through IL-10 to CD11c^+^ APCs resident in or migrating from the lung to suppress the atypical IFN-γ response to HDM. This is reminiscent of early studies demonstrating IL-10 suppression of T_H_1 polarization through direct action on APCs *in vitro* and in nonallergic responses.^14^, ^15^, ^59^ In contrast to the increases in IFN-γ production, which were observed across all models of IL-10 perturbation, HDM-driven IL-17A production and airway neutrophilia were only robustly increased when T cell–derived IL-10 was deleted. Because IL-17A can promote neutrophilic inflammation in the lung,^60^, ^61^ it seems likely that T cell–derived IL-10 suppresses an HDM-induced IL-17A-neutrophil axis partially independently of IL-10Rα on CD11c^+^ myeloid cells, possibly by direct signaling to IL-10R^+^ T_H_17 cells.^62^ However, enhanced neutrophilia in *Il10*^ΔCD4^ mice was partially reversed by αIFN-γ, suggesting that neutrophilia in this context depends on multiple signals, including IFN-γ.

Reciprocal cross-regulation of T_H_2 and T_H_17 responses in asthmatic patients has been suggested based on data in human cells and mouse models,^5^ and it is possible that the increased IL-17A and decreased T2 cytokine expression observed in HDM-treated *Il10*^ΔCD4^ mice reflects such a relationship downstream of IL-10 regulation. However, IL-17A and IL-13 responses can be increased in parallel in asthmatic lungs,^9^ and IL-17A can enhance IL-13 signaling synergistically through signal transducer and activator of transcription 6.^63^ In addition, the role of IL-17A in asthma pathogenesis is incompletely understood, with variable reports of association with disease parameters.^4^, ^64^, ^65^ Indeed, although numbers of both IFN-γ^+^ and IL-17A^+^ T_H_ cells were increased in lungs of patients with severe asthma and a mouse model of severe asthma, only knockout of IFN-γ, but not the IL-17A receptor, was sufficient to improve lung function in these mice.^9^

Although T cell–restricted IL-10 deletion did not increase AHR in our model, we observed enhanced epithelial pathology and airway albumin and uric acid levels in which IL-10 signaling from T cells to CD11c^+^ myeloid cells was disrupted, which was reduced by therapeutic IFN-γ blockade, supporting a pathogenic role for IFN-γ during atypical immunity to inhaled HDM in this context. Increased BAL fluid albumin concentrations can indicate exudation of plasma into the airways without decreased epithelial barrier integrity.^66^ However, concomitant increases in epithelial denudation and loss of uniformity, along with the established epithelial damage-associated molecular pattern uric acid,^44^ collectively support reduced epithelial integrity in our models of IL-10 disruption. IFN-γ can directly disrupt the integrity of human sinus epithelium *in vitro* by promoting apoptosis and decreasing tight junction protein expression,^67^, ^68^ suggesting that epithelial disruption downstream of IFN-γ in our studies might reflect direct effects of this cytokine. It is notable that CD4^+^ T cells were present in increased numbers in the airway lumens of these mice, positioning them optimally for delivering cytokine to the apical side of the epithelium.

It remains to be determined precisely which CD11c^+^ cells and in which temporal and spatial contexts receive IL-10 signals to suppress IFN-γ responses to HDM. This is challenging to dissect with available tools because CD11c-Cre targets both AMs and DCs,^53^, ^69^ whereas LysM-Cre, which is used to broadly target myeloid cells, has variable efficacy in AMs^69^ and affects off-target populations, such as neutrophils.^53^ A recent study showed CD11c-Cre–driven but not LysM-Cre–driven *Il10ra* knockout to prevent induction of tolerance to aeroallergen by using subcutaneous immunotherapy, leading to the conclusion that DCs were the critical IL-10R–expressing cells.^70^ However, this study used peripheral sensitization with an adjuvant, in which involvement of AMs and DCs will almost certainly differ from our mucosal sensitization model. Nonetheless, because T cell–derived IL-10 can suppress T_H_1 responses by preventing IL-12 production from DCs,^59^ it is possible that the absence of such T cell–DC cross-talk in mLNs contributes to the increased IFN-γ responses observed in our models of IL-10 disruption.

Our data strongly support a role for local APCs in perpetuating the IFN-γ response to HDM in which the T-cell–myeloid IL-10 axis is disrupted because AMs and moDCs, both important local influencers of HDM responses,^27^, ^46^ were dysregulated in these mice. Moreover, IL-10 disruption increased expression of the T_H_1 cell chemokines CXCL9 and CXCL10^51^ in lungs, derived at least in part from dysregulated AMs, although contribution from other key pulmonary chemokine producers, such as epithelial cells^71^ and moDCs,^27^ is also possible. Increased lung moDC numbers and CXCR3 ligand expression were both reduced by therapeutic IFN-γ blockade, suggesting that they arise downstream of an initial IFN-γ/IL-10 imbalance. Therefore we propose a model in which a T cell–myeloid IL-10 axis restrains IFN-γ responses to inhaled HDM, disruption of which creates an initial IFN-γ/IL-10 imbalance that unleashes a feedforward loop of dysregulated AMs, moDCs, and CXCR3 ligand production to amplify the IFN-γ response. Reliance on local factors is supported by the fact that IFN-γ^+^ T_H_ cells were more abundant in the lungs but not mLNs of *Il10*^ΔCD4^ mice and that inhaled αIL-10R was sufficient to augment the IFN-γ response to HDM. A CXCR3-dependent amplificatory mechanism also aligns with the association of airway CXCL10 expression with an IFN-γ–high asthma phenotype in human subjects and mice.^7^

Our data highlight the importance of defining the cellular source of IL-10 *in vivo* and examining the regulation of different T_H_ cell subsets after allergen inhalation. Increases in both T2 and T17 cytokine production, as induced in our mouse model, have been observed in the lungs of patients with severe asthma,^9^, ^72^ as have increased IFN-γ responses.^7^, ^8^, ^9^, ^10^ However, none of these studies examined IL-10 levels or function alongside proinflammatory cytokines, despite impaired IL-10 production being implicated in pediatric and adult patients with severe asthma.^22^, ^23^, ^24^ For the first time, our data present a pathogenic mechanism arising from disequilibrium between IL-10 and IFN-γ in the allergic lung and suggest that insufficient IL-10 signaling could contribute to increased IFN-γ responses in patients with severe asthma who might otherwise display a mixed T2/T17 phenotype. Our data might explain in part how heterogeneous phenotypes can occur in certain patients after allergen exposure. Therefore further investigation of the balance between IFN-γ and IL-10 in patients with severe asthma might provide novel insight into disease mechanisms and therapeutic opportunities.

**Key messages**

• HDM inhalation induces IL-10 production from IL-13^+^ and IL-17A^+^ effector T cells in the murine lung.
• IL-10 signaling from T cells to CD11c^+^ myeloid cells limits pathogenic IFN-γ–dependent immunity to HDM.

## Methods

### Tissue processing

Mice were euthanized by exsanguination by cardiac puncture under terminal anesthesia and blood centrifuged for 5 minutes at 12,000*g* in Microtainer Serum Separator Tubes (BD, Franklin Lakes, NJ) to obtain serum. For standard analysis or BAL fluid, the airways were flushed 3 times with 0.4 mL of PBS, pooled fractions were centrifuged, cells were suspended in complete RPMI (cRPMI; RPMI 1640 plus 10% FBS and 1% penicillin and streptomycin; all from Thermo Fisher Scientific), and fluid was stored at −80°C for analysis. For AM sorting, airways were flushed 7 times with 1 mL of 5 mmol/L EDTA in PBS (Thermo Fisher Scientific).

Single-cell lung and mLN suspensions were obtained by passing tissue through 70-μm cell strainers and suspending in cRPMI. Left lungs were minced and incubated in 150 μg/mL collagenase D and 25 μg/mL DNase I (Roche, Basel, Switzerland) at 37°C for 1 hour before dissociation and were subsequently subjected to erythrocyte lysis in ammonium chloride buffer. Right middle lung lobes for mediator analysis were flash-frozen in liquid nitrogen before homogenizing in HBSS (Thermo Fisher Scientific) in the presence of cOmplete protease inhibitor cocktail (Roche). Right postcaval lung lobes for RNA analysis were preserved in RNAlater (Sigma-Aldrich) until analysis. Superior right lobes for histology were inflated with PBS through a major airway and fixed in 10% buffered neutral formalin (Leica Biosystems, Wetzlar, Germany) before processing and staining with either hematoxylin and eosin or periodic acid–Schiff (PAS).

### HDM stimulation of cell suspensions

Lung or lymph node cells (2 × 10^5^) were incubated at 37°C in 200 μL of cRPMI containing 50 μg/mL HDM in 96-well round-bottom cluster plates. Supernatants were collected by means of centrifugation after 4 days.

### Flow cytometry and cell sorting

Up to 2 × 10^6^ cells were stained with Fixable Blue Live/Dead Stain (Thermo Fisher Scientific) at room temperature for 30 minutes, followed by antibodies to extracellular antigens at 4°C for 20 minutes in the presence of mouse Fc Block (clone 2.4G2; BD Biosciences, San Jose, Calif). Cells were washed twice and fixed in eBioscience IC Fixation Buffer (Thermo Fisher Scientific) for 15 minutes at room temperature, except when staining for FoxP3, when the eBioscience Intracellular Fixation & Permablization Buffer Set was used. Where necessary, fixed cells were permeabilized with eBioscience Permeabilization Buffer and stained with antibodies to intracellular antigens at 4°C for 20 minutes before washing twice. When subsequently staining for cytokines, cells were stimulated with PMA and ionomycin for 4 hours at 37°C in the presence of Brefeldin A.

Data were acquired with an LSR Fortessa and FACSDiva software (BD Biosciences) and analyzed with FlowJo software (version 10; TreeStar, Ashland, Ore). All events were gated on live (Live/Dead^−^), singlet CD45^+^ cells before gating leukocyte populations. Fluorescence minus one controls were used, where necessary, to set gates. Fluorescence-activated cell sorting was performed on live BAL cells in the presence of ToPro3 iodide viability dye (Thermo Fisher Scientific) by using a FACSAria (BD Biosciences). AMs were sorted as Topro3^−^ lineage (CD19, CD90.2, NKp46, Ly6G)^−^ CD45^+^ CD64^+^ CD11c^+^ Siglec-F^+^ cells using a 100-μm nozzle to 93% or greater purity.

The following antibodies were used: CD45 (clone 30-F11), CD11c (clone N418), CD64 (clone X54-5/7.1), CD11b (clone M1/70), MHC class II/IA-IE (clone M5/114.15.2), CD103 (clone 2E7), Ly6G (clone 1A8), CD90.2 (clone 53-2/1), CD19 (clone 6D5), NKp46 (clone 29A1.4), Ly6C (clone HK1.4), CD4 (clone RM4-5), CD3e (clone 145-2C11), CD90.1 (clone OX-7), CD44 (clone IM7), CD62L (clone MEL-14), CD69 (clone H1.2F3), IFN-γ (clone XMG1.2), IL-17A (clone TC11-18H10.1), and IL-10 (clone JES-5-16E3; from BioLegend); Siglec-F (clone E50-2440; BD Biosciences); and IL-13 (clone eBio13a) and FoxP3 (clone FJK-16S; Thermo Fisher Scientific).

### Precision-cut lung slices and confocal microscopy

Lungs were inflated with 2% TopVision Low Melting Point Agarose (Thermo Fisher Scientific) through a tracheal cannula, and once solidified, 200-μm transverse slices were prepared from left lungs with a Compresstome VF-300 vibrating microtome (Precisionary Instruments, Greenville, NC). Slices were incubated for 1 hour at 37°C in cRPMI to remove agarose before staining with Brilliant Violet 421–CD4 (clone RM4-5), Alexa Fluor 647–CD11c (clone N418), and phycoerythrin-EpCAM (clone G8.8) antibodies (BioLegend) for 1 hour at 37°C in cRPMI in the presence of Mouse Fc Block. Slices were washed in cRPMI and visualized in 24-well μ-plates (Ibidi, Martinsried, Germany) with an inverted laser-scanning confocal microscope (SP5; Leica Microsystems, Wetzlar, Germany) to acquire 20- to 40-μm Z-stack images of airways. Images of 1024 × 1024 μm field of view were taken under a 20 × objective. Image analysis was performed by using the colocalization tool in Imaris version 8.1 (BitPlane; Oxford Instruments, Abingdon, United Kingdom), with a cutoff of 10 μm for colocalization.

### Airway mucus scoring

All complete airways (≥5) in PAS-stained sections were scored on a 0- to 4-point scale, as previously described,^E1^ with 1 point awarded per 25% of airway epithelial cells positive for PAS and mean scores reported per mouse.

### RNA extraction and quantitative PCR

Lung lobes or cell pellets were homogenized in RLT buffer plus 1% β-mercaptoethanol, and RNA was extracted by using RNeasy Plus Mini (lung) or Micro (cell pellets) kits (Qiagen, Hilden, Germany). Lung RNA (1 μg) and approximately 0.1 μg of cell pellet RNA was converted to cDNA by using the Applied Biosystems High-Capacity Reverse Transcription Kit (Thermo Fisher Scientific) or GoScript Reverse Transcriptase System (Promega, Madison, Wis), respectively, in both cases by using random primers. Quantitative PCR was performed with a Viia7 instrument and TaqMan Fast Advanced Master Mix and the following off-the-shelf assays from Thermo Fisher Scientific: *Gapdh* (Mm99999915_g1), *Actb* (Mm00607939_s1), *Arg1* (Mm00475988_m1), *Chil3* (Mm00657889_mH), *Nos2* (Mm00440502_m1), *Il4* (Mm00445259_m1), *Il5* (Mm00439646_m1), *Il13* (Mm00434204_m1), *Il17a* (Mm00439618_m1), *Ifng* (Mm01168134_m1), *Muc5ac* (Mm01276718_m1), *Muc5b* (Mm00466391_m1), *Col1a1* (Mm00801666_g1), *Col3a1* (Mm01254476_m1), Fn1 (Mm01256744_m1), *Ccl2* (Mm00441242_m1), *Ccl8* (Mm01297183_m1), *Ccl24* (Mm00444701_m1), *Cxcl9* (Mm00434946_m1), *Cxcl10* (Mm00445235_m1), and *Cxcl16* (Mm00469712_m1). Threshold cycle (Ct) values were determined by using Viia7 software, and relative expression was calculated as 2^−ΔCt^ relative to the mean Ct of the housekeeping genes *Gapdh* and *Actb*. Data are presented either as unadjusted relative expression or fold changes from means of an appropriate negative control group.

### Mediator analysis

IFN-γ and IL-17A levels were measured by using Ready-Set-Go ELISA kits (Thermo Fisher Scientific), IL-13 levels were measured by using the mouse Quantikine ELISA Kit (R&D Systems, Minneapolis, Minn), and albumin levels were measured by using the Bethyl Laboratories (Montgomery, Tex) Mouse Albumin ELISA Kit, according to the manufacturers' instructions. IgE, IgG_1_, and IgG_2a_ were detected by using custom sandwich ELISAs consisting of purified capture antibodies (clones R35-72, A85-3, and R11-89, respectively) and biotinylated detection antibodies (clones R35-118, A85-1, and R19-15, respectively) standardized to purified IgE, IgG_1_, and IgG_2a_ κ isotype control antibodies (all from BD Biosciences) and developed by using streptavidin–horseradish peroxidase (R&D Systems) and TMB substrate (Thermo Fisher Scientific). Uric acid was quantified by using the Amplex Red Uric Acid/Uricase Assay Kit (Thermo Fisher Scientific), according to the manufacturer's instructions. Sample analyte concentrations were determined from standard curves by using a SpectraMax i3X plate reader and SoftMax Pro 7 software (Molecular Devices, San Jose, Calif).
